# miR-23a-5p Alleviates Gouty Inflammation by Targeting Interleukin-17A and Inhibiting NLR Family Pyrin Domain Containing 3 Inflammasome Activation

**DOI:** 10.5152/ArchRheumatol.2025.11198

**Published:** 2025-12-01

**Authors:** Xinzhu Yuan, Changwei Lin, Yanni Zhang, Hongni He, Lingqin Li

**Affiliations:** 1 Department of Nephrology, The Second Clinical Medical College of North Sichuan Medical College, Nanchong Central Hospital, Nanchong, China; 2 Department of Nephrology, Affiliated Hospital of North Sichuan Medical College, Nanchong, China; 3 Department of Gynaecology, The Second Clinical Medical College of North Sichuan Medical College, Nanchong Central Hospital, Nanchong, China; 4 Department of Rheumatology and Immunology, Affiliated Hospital of North Sichuan Medical College, Nanchong, China

**Keywords:** Gouty inflammation, IL-17A, miR-23a-5p, NLRP3 inflammasome, THP-1 cells

## Abstract

**Background/Aims:**

Monosodium urate (MSU) is a key contributor to gout development, primarily by triggering innate inflammatory responses. Given the known role of miR-23a-5p in regulating inflammation, this study aimed to investigate the molecular mechanisms through which miR-23a-5p influences MSU-induced gout inflammation.

**Materials and Methods:**

Monosodium urate was used to model gouty inflammation in THP-1 cells and Sprague–Dawley (SD) rats.In vivo, 15 SD rats were randomly divided into the control, model, model + miR-23a mimic, model + interleukin (IL)-17A, and model + miR-23a mimic + IL-17A groups. In vitro, the model cells were treated with miR-23a mimics, IL-17A, or both. TargetScan predicted that miR-23a-5p might target IL-17A, which was verified using a dual-luciferase reporter system. Subsequently, IL-17A and miR-23a-5p mRNA levels, as well as NLR family pyrin domain containing 3 (NLRP3) inflammasome–related factors (IL-1β, IL-6, NLRP3, IL-18, ASC, and caspase-1), were quantified using quantitative polymerase chain reaction, immunohistochemistry, enzyme-linked immunosorbent assay, and western blotting. Hematoxylin and eosin (H&E) staining was performed to observe pathological changes in rat ankles. Additionally, flow cytometry was conducted to quantify Th17 cells in rat blood.

**Results:**

Both in vivo and in vitro, miR-23a-5p expression was downregulated, whereas IL-17A expression was upregulated in the gout models. The H&E staining revealed that miR-23a-5p mimic treatment alleviated gout symptoms, whereas IL-17A treatment exacerbated these symptoms. Direct interaction between miR-23a-5p and IL-17A was validated using a dual-luciferase reporter assay. Moreover, flow cytometry analysis showed that miR-23a-5p overexpression inhibited Th17 cell differentiation. Additionally, miR-23a-5p suppressed NLRP3 inflammasome activation by targeting IL-17A. Correspondingly, the expression of proinflammatory proteins such as IL-1β, IL-6, NLRP3, IL-18, and caspase-1 was downregulated by miR-23a-5p and upregulated by IL-17A treatment.

**Conclusion:**

This study demonstrated that miR-23a-5p alleviates MSU-induced gouty inflammation by directly targeting IL-17A. Through this regulation, miR-23a-5p suppresses NLRP3 inflammasome activation, highlighting its potential as a therapeutic target for gout.

**Main Points**

- miR-23a-5p directly targets IL-17A to suppress Th17 cell differentiation and inflammatory cytokine release in gout
- Overexpression of miR-23a-5p inhibits NLRP3 inflammasome activation and alleviates MSU-induced gouty inflammation
- miR-23a-5p may serve as a promising therapeutic target for the prevention and treatment of gout

## Introduction

Gout results from tissue damage caused by persistently high uric acid levels and monosodium urate (MSU) crystal deposition in the joints or tendons. Its main clinical manifestations are hyperuricemia and joint pain.^1^ The complex pathogenesis of gout is generally thought to be regulated by proinflammatory cytokines, including tumor necrosis factor (TNF-α), interleukin (IL)-6, IL-18, and IL-1β. The inflammatory response to gout has been reported to be related to the nuclear factor kappa B (NF-κB) pathway,^1-3^ with innate immune pathways being crucial for the pathogenesis of gout. In particular, the activation of the NLR family pyrin domain containing 3 (NLRP3) inflammasome usually leads to the release of proinflammatory cytokines such as IL-1β and IL-18.^4^ Furthermore, recent studies have identified the inhibition of NLRP3 inflammasome formation as a potential therapeutic target for mitigating gout development.^5^ The NLRP3 inflammasome is implicated not only in the activation of the NF-κB signaling pathway but also in apoptosis. Thus, targeting NLRP3 inflammasome activation may serve as a viable strategy for gout treatment.^6^

MicroRNAs (miRNAs) are a family of short noncoding RNAs that function as competitive endogenous RNAs and interact with long noncoding RNAs.^7^ In recent years, miRNAs have been recognized for their roles in regulating gene expression, which is crucial for the pathogenesis and progression of various diseases.^8^ Specifically, miR-23a-5p has been shown to participate in oxidative stress and inflammatory responses.^9,10^ In 2021, miR-23a-5p was found to be significantly downregulated in the serum of patients with rheumatoid arthritis and TNF-α-treated human fibroblast-like synoviocytes (MH7A cells), suggesting its potential as a therapy target for rheumatoid arthritis. It targets the Toll-like receptor 4 (TLR4)/myeloid differentiation primary response 88 (MyD88)/NF-κB pathway, thereby inhibiting the inflammatory process.^11^ Nevertheless, the molecular mechanism by which miR-23a-5p regulates immune control in gout remains unclear.

IL-17 is derived from T helper 17 (Th17) cells (a subset of CD4+ T cells) and typically plays an important role in autoimmune diseases, including rheumatoid arthritis, psoriasis, and inflammatory bowel disease.^12^ The IL-17A neutralizing antibody has recently been shown to alleviate inflammation in mice with gout.^13^ Additionally, multiple studies have demonstrated that IL-17A can activate NLRP3 inflammasome formation.^14,15^

The present study aimed to investigate the molecular mechanisms through which miR-23a-5p influences MSU-induced gouty inflammation. In this study, TargetScan (https://www.targetscan.org/vert_80/) was employed to predict whether IL-17A may be a target gene of miR-23a-5p. It was inferred that miR-23a-5p might target IL-17A to inhibit NLRP3 inflammasome activation and alleviate gouty inflammation. Thus, miR-23a-5p is expected to have wide applications in gout treatment. In this study, the molecular mechanisms were explored based on in vivo and in vitro studies.

## Methods

### Cell Culture and Modeling Monosodium Urate–Induced Gouty Inflammation

The THP-1 cells were purchased from Cell Biosciences (Wuhan, China). Dulbecco’s modified Eagle medium (Invitrogen, USA) was used to culture cells at 37°C in 5% CO_2_. The medium was supplemented with 10% fetal bovine serum (Sigma-Aldrich, USA).

In Experiment 1 (in vitro, n = 3), cells were randomly divided into 4 groups: control, phorbol myristate acetate (PMA) + 50 μg/mL MSU, PMA + 100 μg/mL MSU, and PMA + 200 μg/mL MSU. In this study, THP-1 cells were incubated with 100 ng/mL PMA for 24 hours to differentiate into macrophages. Subsequently, cells were stimulated by 50, 100, and 200 μg/mL MSU for 6 hours to model gout. All animal experimental procedures were approved by the Ethics Committee for Laboratory Animal Care and Use of West China Hospital, Sichuan University (Approval No. 20240828001). Informed Consent was not applicable, as this study did not involve human participants.

### Transfection with miRNA

To overexpress miR-23a, Lipofectamine 2000 in Opti-MEM (Invitrogen) was used to transfect 5 pmol of hsa-miR-23a-5p (RiboBio, China) into THP-1 cells for 48 hours following the manufacturer’s instructions. An NC mimic (RiboBio, China) was used as a control.

In Experiment 2 (in vitro, n = 3), the cells were divided into control, NC mimic (blank), and miR-23a mimic groups. Model cells were stimulated by 100 ng/mL PMA for 24 hours following stimulation with 100 µg/mL MSU for 6 hours.

In Experiment 3 (in vitro, n = 3), the cells were divided into control, model, model + NC mimic, and model + miR-23a mimic groups. Model cells were transfected with an NC mimic or miR-23a mimic.

In Experiment 4 (in vitro, n = 3), the cells were divided into control, model, model + miR-23a mimic, model + IL-17A, and model + miR-23a mimic + IL-17A groups. Model cells were transfected with an miR-23a mimic, 300 μg/mL IL-17A, or both.

### Gout Rat Modeling and Drug Treatment

Fifteen Sprague–Dawley (SD) rats were obtained from Chengdu Dashuo Co. In Experiment 4 (in vivo, n = 3), rats were randomly divided into control, model (gout), miR-23a mimic, IL-17A, and miR-23a mimic + IL-17A groups. The acute gout rat model was established via unilateral injection of 200 MSU solution (2.5 g/100 mL) into the ankle joint cavity, and the success of modeling was defined by contralateral bulging of the joint capsule. Furthermore, 10 μL of miR-23a mimic and/or IL-17A was injected into the ankle joint cavity of the miR-23a mimic, IL-17A, and miR-23a mimic + IL-17A groups at 3, 5, 7, and 10 days, respectively. An equal amount of saline was used in both the control and model groups.

### Cell Counting Kit-8 assay for cell viability

Cell viability was defined using the cell counting kit-8 (CCK-8) assay. THP-1 cells were cultured in logarithmic growth phase. Pre-warmed medium was used to prepare the cell suspension, and the cell concentration was adjusted to 1 × 10^5^ cells/mL for the cell seed. Each well of the 96-well cell culture plate was seeded with 100 μL and inoculated. After the cells adhered, the original medium was aspirated. The THP-1 cells were treated in cell culture, as described previously. On the next day, 10 μL CCK-8 solution was added to each well and incubated for 4 hours. Finally, the absorbance of each well was measured at 450 nm.

### Hematoxylin and Eosin and Immunohistochemistry Staining

Rat ankle joint tissues were fixed with 4% paraformaldehyde and placed in 10% ethylenediaminetetraacetic acid solution (YE0105, BIOBOMEI, China) to complete tissue decalcification. Decalcified tissues were dehydrated with alcohol, embedded in paraffin wax, and sliced. The slices were stained using an hematoxylin and eosin staining kit (Beyotime, Beijing, China). For immunohistochemistry (IHC) staining, the slices were incubated in 3% hydrogen peroxide at room temperature and protected from light for 25 minutes to block endogenous peroxidase activity. The samples were washed 3 times with phosphate-buffered saline (PBS) and blocked with bovine serum albumin (BSA). The slices were incubated with the primary antibody against apoptosis-associated speck-like protein containing a CARD (ASC) (1 : 100, A1170; ABclonal) at 4°C overnight, followed by incubation with the secondary antibody (1 : 100, GB22303, Servicebio) at 37°C for 30 minutes. Images were analyzed using a BA400 digital trinocular video microscope and Halo 101-WL-HALO-1 data image analysis system.

### Detection of miR-23a-5p and Interleukin-17A

Quantitative real-time polymerase chain reaction (qRT-PCR) was performed to investigate the mRNA expression levels of miR-23a-5p and IL-17A. Based on the manufacturer’s instructions, the TRIzol reagent was used to extract total RNA (Invitrogen, Carlsbad, CA, USA), and cDNA was obtained using the miScript II RT Kit (Qiagen). The samples were analyzed using the SYBR Green assay (Vazyme, China), with β-actin and U6 serving as the control. The thermocycling parameters were 95°C for 10 minutes, 95°C for 15 seconds, and 60°C for 1 minute for 40 cycles, whereas the dissociation curve parameters were 95°C for 15 seconds, 60°C for 15 seconds, and 95°C for 15 seconds. The Roche LightCycler 480 Real-Time PCR System was applied for the experiment following the protocol, and the 2^−ΔΔCt^ method was used for analysis. Table 1 presents the primer sequences.

### Enzyme-Linked Immunosorbent Assay

Enzyme-linked immunosorbent assay (ELISA) was performed to quantify the IL-1β and IL-6 levels. Quantitatively, ELISA plates were coated overnight with purified IL-1β and IL-6 antibodies in carbonate-bicarbonate buffer at 4°C. Subsequently, 200 μL blocking buffer BSA was used to block the ELISA plates at 37°C for 1 hour. Next, samples were added and incubated at 37°C for 2 hours. After 3 PBS washes, the samples were incubated with horseradish peroxidase-conjugated IL-1β and IL-6 antibodies at 37°C for 1 hour and then washed 3 times. Finally, the substrate was added and the mixture was incubated for 10 minutes, after which the enzyme reaction was terminated. Optical density was measured at 450 nm. Each sample was tested in triplicates.

### Western Blotting

The RIPA buffer (Beyotime, China) was used to lyse the samples. The lysates were separated using sodium dodecyl sulfate–polyacrylamide gel electrophoresis and transferred to polyvinylidene difluoride membranes. The membranes were blocked with 5% non-fat milk at room temperature for 1 hour. Next, primary antibodies against NLRP3 (1 : 1000, no. 19771-1-AP; Proteintech, China), caspase-1 (1 : 1000, no. 22915-1-AP; Proteintech, China), IL-18 (1 : 1000, no. A1115; ABclonal, Wuhan, China), IL-17A (1 : 1000, no. ab218013; Abcam, UK), and β-actin (1 : 100000, no. AC026; ABclonal, Wuhan, China) were used to incubate with the membrane overnight at 4ºC, followed by incubation with goat anti-rabbit IgG H&L (1 : 5000, Abcam, no. ab6721, UK) or goat anti-mouse IgG H&L (1 : 5000; Abcam, no. ab6789, UK) for 1 hour. Enhanced chemiluminescence detection kits (Proteintech, China) and ImageJ software (Bethesda, MD, USA) were used to detect protein expression.

### Dual-Luciferase Assay

HEK-293T cells were seeded in 48-well plates. When cells were grown in the logarithmic phase, they were transfected with 5 pmol hsa-miR-23a-5p, NC mimic, and 160 ng hsa-IL-17A-3UTR for 48 hours. To determine the luciferase reporter activities, the samples were collected and lysed using the Dual-Glo® Luciferase Assay System (Promega, USA).

### Flow Cytometry

Flow cytometry was used to quantify the Th17 cells. Rat blood cells were lysed using erythrocyte lysate (G2015-500ML, Servicebio, China), and the cell sediment was collected for staining with FITC anti-rat CD4 (201505, Biolegend, USA) and IL-17A monoclonal antibody PE IL-17A (12-7177-81, Thermo Fisher, USA) incubation. Data were analyzed using the Cytoflex flow analyzer (Beckman, USA) and CytExpert software (Beckman, USA).

### Statistical Analysis

Statistical analyses for all experiments were performed using GraphPad Prism software version 9.5.1 (GraphPad Software; San Diego, CA, USA). Data were expressed as means ± standard deviation. One-way analysis of variance (ANOVA) was used for the comparison among multiple groups. The difference between 2 groups was analyzed using Student’s *t*-test. Statistical significance was set at *P *< .05.

## Results

### Effect of miR-23a-5p on NLR Family Pyrin Domain Containing 3 Inflammasome in Monosodium Urate–Induced THP-1 Cells

**Figure 1.**
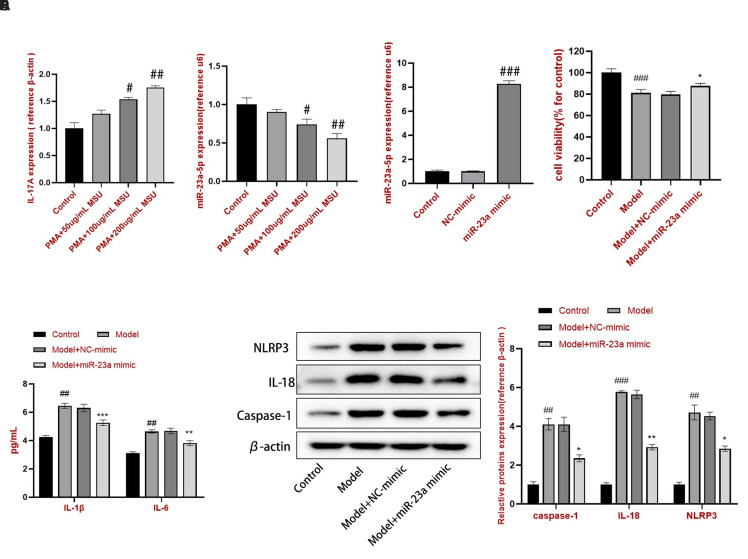
Effect of miR-23a-5p overexpression on apoptosis and inflammation in the MSU-induced gouty inflammation model. qRT-PCR was performed to detect the (A) IL-17A and (B) miR-23a-5p expression and to quantify the (C) miR-23a-5p expression. (D) Cell viability. (E) ELISA was performed for IL-1β and IL-6 content. (F) Western blotting was performed to detect caspase-1, IL-18, and NLRP3 protein expression. Line charts and bars represent mean ± SD from 3 independent experiments. ^##^*P* < .01 and ^###^*P* < .001 vs. the control group. ^*^P < .05, ^**^P < .01, and ^***^P < .001 vs. the model + NC mimic group.

The results of qRT-PCR performed to detect IL-17A and miR-23a-5p expression indicated that 100 and 200 μg/mL MSU significantly increased the IL-17A expression (Figure 1A) and decreased the miR-23a-5p expression (Figure 1B) compared with those in the control group. The NC mimic and miR-23a-5p mimic were transfected into THP-1 cells. The miR-23a-5p expression was upregulated in the miR-23a mimic group (Figure 1C), suggesting that miR-23a mimic transfection was successful. Monosodium urate reduced cell viability; however, the miR-23a mimic reversed this effect (Figure 1D). Furthermore, IL-1β and IL-6 contents were detected by ELISA. Monosodium urate stimulated THP-1 cells to secrete IL-1β and IL-6; however, the release of IL-1β and IL-6 was downregulated when miR-23a-5p was overexpressed in MSU-induced THP-1 cells (Figure 1E). Furthermore, NLRP3 inflammasome-related proteins, including caspase-1, IL-18, and NLRP3, were detected by western blotting (Figure 1F). Compared to the control group, the model group showed higher protein expression of caspase-1, IL-18, and NLRP3. The results revealed that NC mimic transfection caused no obvious changes compared with the model group. Nonetheless, compared to the model + NC mimic group, the model + miR-23a mimic group showed lower protein expression of caspase-1, IL-18, and NLRP3.

### miR-23a-5p Bound Interleukin-17A to Inhibit the Proliferation and Inflammatory Cytokine Secretion in THP-1 Cells

**Figure 2.**
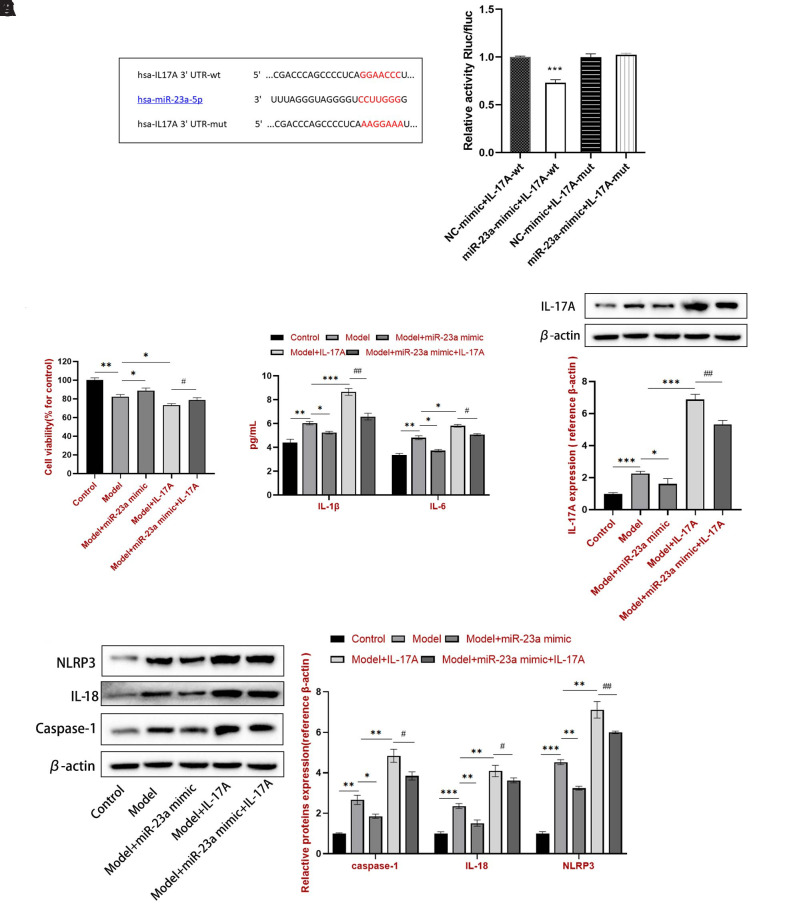
miR-23a-5p bound IL-17A to inhibit NLRP3 inflammasome activation in THP-1 cells. (A) The putative miR-23a-5p binding sites in the IL-17A sequence. (B) miR-23a mimics or NC mimics and pSI-Check2-ATG7-3’UTR plasmid were co-transfected into HEK-293T cells. ***P < .001 vs. the NC mimic + IL-17A-wt group. (C) Cell viability. (D) ELISA was performed for IL-1β and IL-6 content. Western blotting was conducted to measure protein expression, including (E) IL-17A and (F) caspase-1, IL-18, and NLRP3. ^*^P < .05, ^**^P < .01, and ^***^P < .001 vs. the model group. ^##^*P* < .01 and ^###^*P* < .001 vs. the model + IL-17A group.

In this study, the relationship between miR-23a-5p and IL-17A expression was predicted using the online tool TargetScan (Figure 2A). The IL-17A 3′UTR-wide type (wt) and IL-17A 3′UTR-mutation (mut) luciferase reporters were constructed with firefly luciferase, and the luciferase assay was conducted. Compared with the NC mimic group, the miR-23a-5p mimic prominently reduced the luciferase activity of cells transfected with IL-17A 3′UTR‑wt; in contrast, no significant difference in the IL-17A 3′UTR‑mut group was observed (Figure 2B). These results suggested that miR-23a-5p directly targeted IL-17A. To further explore this, cell viability was determined using the CCK-8 assay, and the results indicated that the addition of miR-23a-5p increased cell viability compared to the model + IL-17A group (Figure 2C). The miR-23a-5p mimic could rescue IL-17A-treatment caused IL-1β and IL-6 upregulation (Figure 2D). Furthermore, western blotting analysis revealed that MSU increased the IL-17A (Figure 2E), caspase-1, IL-18, and NLRP3 levels (Figure 2F). These effects were reinforced by IL-17A, but were alleviated by miR-23a-5p mimic. All proteins were downregulated in the model + miR-23a mimic + IL-17A group compared to the model + IL-17A group, suggesting that miR-23a-5p may target IL-17A to inhibit NLRP3 inflammasome activation.

### miR-23a-5p Targets Interleukin-17A to Alleviate Gout in Sprague–Dawley Rats

**Figure 3.**
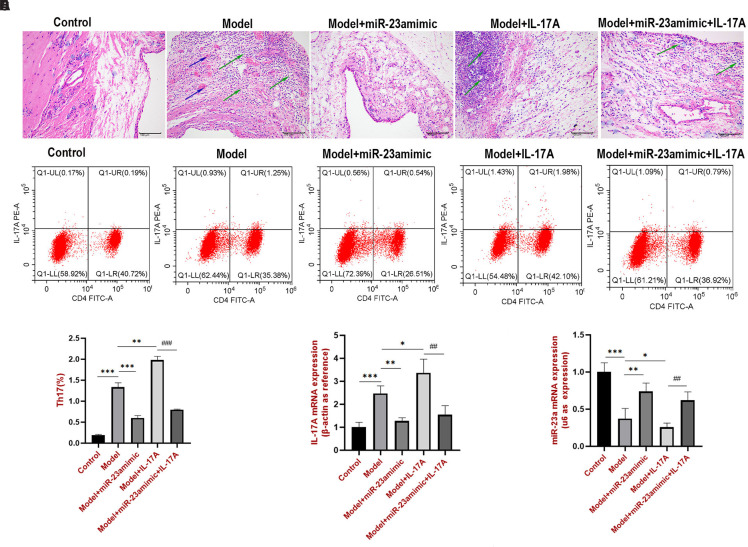
miR-23a-5p targeted IL-17A to alleviate gout in SD rats. (A) H&E staining of the knee. (B, C) Th17 cell subsets were detected by flow cytometry. (D) IL-17A mRNA expression. (E) miR-23a-5p was quantified by qPCR. ^*^P < .05, ^**^P < .01, and ^***^P < .001 vs. the model group. ^##^*P* < .01 and ^###^*P* < .001 vs. the model + IL-17A group.

To further demonstrate the miR-23a-5p effects in vivo, a gout rat model was established via unilateral injection of MSU. The model rats were treated with miR-23a mimic, IL-17A, or both. Compared to those of the control group, the ankle joint tissues of the remaining 4 rat groups exhibited varying degrees of necrosis, edema, inflammatory cell infiltration, and vascular hyperplasia in the subsynovial lining layer (Figure 3A). The IL-17A group showed aggravated lesions, whereas the miR-23a mimic group had attenuated lesions compared to the model group. The IL-17A + miR-23a mimic group exhibited fewer lesions than the IL-17A group. The Th17 cell subtypes (Figure 3B and C) and IL-17A (Figure 3D) were detected separately. These results demonstrated that the miR-23a mimic significantly reduced Th17 cell differentiation and IL-17A expression. In addition, miR-23a expression in the ankle joints of rats was verified using qPCR; miR-23a mRNA was upregulated by the miR-23a mimic in rats with gout, whereas IL-17A was downregulated (Figure 3E).

### miR-23a-5p Targeted Interleukin-17A to Alleviate Gout by Inhibiting the NLR Family Pyrin Domain Containing 3 Inflammasome in Sprague–Dawley Rats

**Figure 4.**
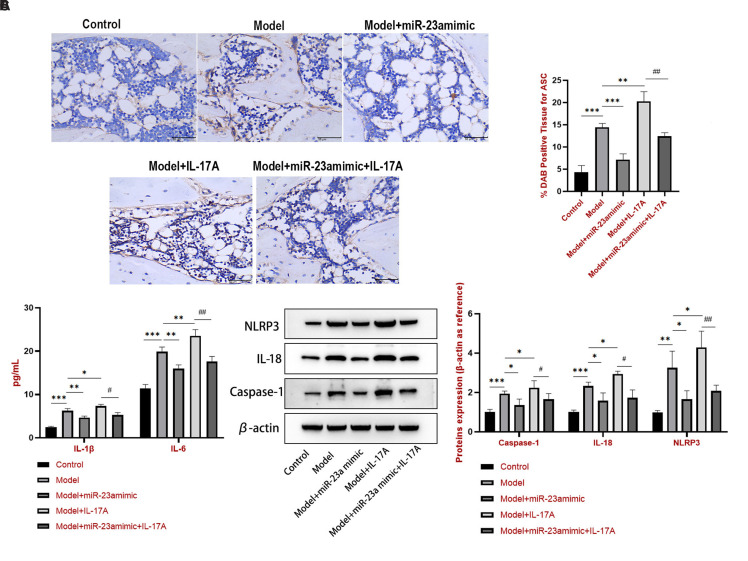
miR-23a-5p targets IL-17A to alleviate gout by activating the NLRP3 inflammasome in SD rats. (A) IHC was conducted to detect ASC. (B) ELISA was performed for IL-1β and IL-6 content. (C) caspase-1, IL-18, and NLRP3 protein expression was measured by western blotting. ^*^P < .05, ^**^P < .01, and ^***^P < .001 vs. the model group. ^#^*P* < .01, ^##^*P* < .01, and ^###^*P* < .001 vs. the model + IL-17A group.

ASC is another marker of NLRP3 inflammasome. Thus, IHC was used to detect ASC protein expression (Figure 4A), ELISA to quantify the release of IL-1β and IL-6 cytokines (Figure 4B), and western blotting to measure caspase-1, IL-18, and NLRP3 expression (Figure 4C). The results indicated MSU-induced upregulation of ASC, IL-1β, IL-6, caspase-1, IL-18, and NLRP3. Compared to the model group, the IL-17A group showed intensified expression of these proteins, whereas the miR-23a mimic group exhibited diminished expression. Importantly, miR-23a mimic alleviated the IL-17A-induced upregulation of ASC, IL-1β, IL-6, caspase-1, IL-18, and NLRP3.

## Discussion

Gout development is closely associated with the activation of the innate immune system and the concentration of uric acid, which eventually forms urate deposits in the joint cavities, triggering pain. Raucci et al^13^ reported the beneficial effects of IL-17 inhibition in attenuating joint swelling and leukocyte infiltration. Additionally, accumulating evidence suggests that MSU crystals stimulate the innate immune response and upregulate the expression of proinflammatory cytokines such as IL-17A, IL-6, and IL-1β.^16,17^ In the present study, IL-17A, IL-6, and IL-1β were increased in the MSU-induced THP-1 cells, which was consistent with the findings of previous studies. Moreover, MSU exposure led to the downregulation of miR-23a-5p in these cells, whereas miR-23a-5p overexpression enhanced cell viability. These results suggest that targeting IL-17A and enhancing miR-23a-5p expression are promising therapeutic approaches for gout. Nonetheless, the underlying molecular mechanisms remain unclear.

Recent studies have highlighted the critical roles of miRNAs in the development and progression of various diseases. Several miRNAs have been reported to be either upregulated or downregulated in gout, contributing to disease pathogenesis.^18,19^ For example, miR-3146 mediates NETs formation and may play a role in the pathogenesis of gout.^18^ miR-146a overexpression may downregulate IL-1β expression in MSU-induced THP-1 cells.^20^ Importantly, miR-23a-5p has been shown to target Runx2, which is involved in osteogenic differentiation.^21^ However, its role in gout-related inflammation remains unknown.

Interleukin-17A has been demonstrated to recruit neutrophils and induce chemokines such as IL-1, IL-6, IL-18, and TNF-α.^22,23^ As the study demonstrated, the expression of IL-18 was downregulated significantly by miR-23a-5p overexpression. It was found that miR-23a-5p targeted IL-17A to inhibit Th17 cell differentiation. Furthermore, IL-17A was identified as a direct target of miR-23a-5p. miR-23a-5p overexpression in MSU-induced THP-1 cells led to the downregulation of IL-17A, which is known to activate the NF-κB signaling pathway and promote the secretion of proinflammatory cytokines such as IL-1β and IL-6. Expressions of IL-1β and IL-6 both decreased with miR-23a-5p. These findings suggest that miR-23a-5p is a promising therapeutic target for modulating inflammation in gout.

The NLRP3 inflammasome activation involves the assembly of NLRP3, pro-caspase-1, and ASC.^24^ In this study, the expression levels of caspase-1, IL-1β, and NLRP3 were significantly upregulated in both THP-1 cells and gout SD rats; however, the miR-23a-5p mimic markedly reduced protein expression. This suggests that miR-23a-5p inhibits NLRP3 inflammasome activation. One previous study reported that the loss of NLRP3 inflammasome activity in neutrophils could alleviate gout symptoms.^25^ Another study found that MSU formation exacerbated NLRP3 inflammasome activation, thereby promoting gout development.^26^ Further animal experiments demonstrated that IL-17A could aggravate MSU-induced NLRP3 inflammasome formation and that the miR-23a mimic could reverse this effect. These findings suggest that miR-23a-5p targets IL-17A to suppress NLRP3 inflammasome activation, offering a potential mechanistic pathway for gout treatment. A recent study on colorectal cancer showed that miR-23a-5p could bind to P14AS to inhibit TNF-NF-κB signaling and decrease IL-6 and IL-8 levels.^27^ Another study suggested that miR-23a-5p agomir injection effectively reduced airway inflammation in mice with chronic obstructive pulmonary disease.^28^ Although in a different context, these findings support the view that miR-23a-5p regulates inflammation, consistent with its anti-inflammatory role in MSU-induced gout.

This study has certain limitations, particularly its small sample size, which might have reduced the statistical power and restricted the generalizability of the results. However, the observed trends were consistent and biologically significant.

The findings suggest that miR-23a-5p may serve as a potential therapeutic target for controlling MSU-induced gout inflammation. By directly targeting IL-17A, miR-23a-5p plays a pivotal role in regulating NLRP3 inflammasome activation both in THP-1 cells and gout rats. This study provides new insights into the molecular mechanisms underlying the pathogenesis of gout and offers a valuable reference for the development of novel treatment strategies.

## Figures and Tables

**Table 1.** Primers of qRT-PCR Analysis

Gene	Forward Primer 5′-3′	Reverse Primer 5′-3′
miR-23a-5p	GGGGUUCCUGGGGAUGGGAUUU	Universal primers (miScript SYBR Green PCR kit)
IL-17A	TGTGCCTGATGCTGTTGCTGCTACTG	GGTCCTCATTGCGGCTCAGAGTCCA
β-actin	GAAGATCAAGATCATTGCTCC	TACTCCTTGCTGATCCA
U6	CAGCTGCT TCCTCTAGAGC	CAAATCCCAGAAGTGTGCCA

## Data Availability

The data that support the findings of this study are available on request from the corresponding author.
